# Epicardial Adipose Tissue: A Potential Target to Improve Left Ventricular Diastolic Dysfunction

**DOI:** 10.31083/RCM39224

**Published:** 2025-09-25

**Authors:** Chun-Qiong Ran, Wen-Tao He

**Affiliations:** ^1^Division of Endocrinology, Department of Internal Medicine, Tongji Hospital, Tongji Medical College, Huazhong University of Science and Technology, 430030 Wuhan, Hubei, China; ^2^Hubei Clinical Medical Research Center for Endocrinology and Metabolic Diseases, 430030 Wuhan, Hubei, China; ^3^Branch of National Clinical Research Center for Metabolic Diseases, 430030 Wuhan, Hubei, China

**Keywords:** epicardial adipose tissue, left ventricular diastolic dysfunction, assessment, pathophysiology, medications

## Abstract

Left ventricular diastolic dysfunction (LVDD) can progress to heart failure, a condition associated with diminished quality of life as well as high mortality. Meanwhile, timely diagnosis and effective treatment of LVDD rely on a thorough understanding of the pathogenesis involved in LVDD. Echocardiography and cardiac magnetic resonance are the primary imaging modalities for evaluating left ventricular diastolic function. Several strands of evidence indicate that increased epicardial adipose tissue (EAT) correlates with LVDD in various clinical settings, such as hypertension, coronary artery diseases, diabetes, and obesity. Conversely, therapeutic strategies aimed at reducing EAT may improve the restoration of diastolic function. Some interventions have shown promise in decreasing EAT, including medications (hypoglycemic and hypolipidemic agents), lifestyle modifications (diet and exercise), and bariatric surgery. Notably, these interventions have concurrently been linked to improvements in diastolic parameters. This review compiles recent advancements in the clinical evaluation of LVDD to elucidate the pathophysiological and therapeutic roles of EAT in LVDD.

## 1. Introduction

Left ventricular diastolic dysfunction (LVDD) is commonly defined as impaired myocardial relaxation with normal ejection fraction and the absence of heart failure symptoms [1]. Its development and progression correlate with various comorbidities, including hypertension, obesity, diabetes, and others [2]. If not properly treated, LVDD will evolve into heart failure, leading to increased mortality [3]. Various mechanisms have been proposed to explain relaxation abnormalities, including disruptions in energy metabolism, calcium ion homeostasis, and myofilament function, as well as inflammation and oxidative stress [4, 5, 6]. Nonetheless, these mechanisms inadequately account for the pathophysiological processes underlying the occurrence and progression of diastolic dysfunction. Over the past decades, converging evidence has demonstrated that epicardial adipose tissue (EAT) is an independent risk factor for LVDD.

EAT, located between the myocardium and visceral pericardium, shares the same microcirculation with adjacent myocardial tissue [7, 8]. Considering its contiguity with both myocardium and coronary arteries, EAT has attracted considerable interest for its potential involvement in the pathophysiology of LVDD. For example, increased EAT has been associated with detrimental alteration in cardiac structure and diastolic function in patients with prediabetes [9]. Besides, it has been shown that EAT volume index was a robust predictor for LVDD in patients with chronic coronary syndrome [10]. But the specific role of EAT in this process has not been fully elucidated. Additionally, it has yet to be established whether EAT can serve as a potential therapeutic target for LVDD, with the aim of preventing or delaying the occurrence of heart failure.

At present, there is a lack of comprehensive review discussing mechanisms by which EAT may be implicated in LVDD pathophysiology and the possible benefits of reducing EAT in the management of LVDD. Herein, we review current knowledge about the clinical assessment methods of LVDD, and potential therapeutic roles of EAT for LVDD in various clinical settings.

## 2. Assessment Methods of Diastolic Function

### 2.1 Echocardiography

Currently, echocardiography is the primary non-invasive imaging modality for evaluating left ventricular (LV) diastolic function. LV diastole typically consists of four phases: isovolumic relaxation, rapid filling, diastasis, and atrial systole [11]. Diastolic function assessment integrates multiple echocardiographic parameters. Tissue Doppler imaging (TDI) measures the LV regional lengthening velocity to derive the mitral annular early diastolic velocity (e’), a critical parameter reflecting LV relaxation and restoring forces, which is inversely related to the relaxation time constant, tau [12]. In addition, the velocity captured during the late (a’) phase of diastole can also be obtained using TDI, with a decrease in the e’/a’ ratio serving as an indicator of diastolic dysfunction [13]. The early diastolic transmitral flow velocity (E) indicates the pressure gradient between the left atrial (LA) and LV during early diastole, influenced by both rate of LV relaxation and LA pressure. Conversely, the late diastolic velocity (A) reflects the pressure gradient during atrial contraction, determined by LV compliance and LA function [12]. The E/A ratio classifies LV filling patterns. The E/e’ ratio, a robust and reproducible indicator, provides reliable assessment of diastolic function, with an average E/e’ >14 indicating elevated LV filling pressure [13]. Additionally, the isovolumetric relaxation time and the deceleration time of the early transmitral flow velocity are fundamental parameters that provide insights into relaxation and restoring forces [11]. Other indices, including tricuspid regurgitation (TR) systolic jet velocity, maximum LA volume index (LAVI), and pulmonary venous flow velocities (both systolic and diastolic), are also employed to evaluate LV filling pressure [12, 13]. Recent advances incorporate strain imaging techniques into functional assessment. Speckle tracking strain imaging has facilitated the evaluation of twisting movement and LV untwisting. Novel indices, such as the global LV longitudinal diastolic strain rate during isovolumetric relaxation and early diastole, have been shown to effectively reflect LV diastolic function [14, 15]. Furthermore, diastolic stress echocardiography, utilizing pharmacological or exercise-induced stress, has emerged as a valuable method for detecting subclinical diastolic dysfunction [16, 17].

In patients with reduced left ventricular ejection fraction (LVEF) and/or any of the prior myocardial diseases, those with an E/A ratio ≤0.8 and E velocity ≤50 cm/s are graded I diastolic dysfunction with normal or low left atrial pressure. When the E/A ratio is ≥2, LA pressure is increased and denotes grade III diastolic dysfunction. For patients with an E/A ratio <0.8 and a peak E velocity >50 cm/s, or an E/A ratio between 0.8 and 2, additional parameters such as TR velocity, E/e’ ratio, and LAVI are assessed to determine elevated left atrial pressure. Grade II diastolic dysfunction is diagnosed if at least half of these additional parameters meet abnormal criteria [12, 18]. For patients with normal LVEF and no myocardial disease, diastolic dysfunction is defined using 4 recommended echocardiographic variables (or a majority of the 4 parameters) and their abnormal cutoff values. The variables and cutoff values are as follows: annular e’ velocity (septal e’ <7 cm/s, lateral e’ <10 cm/s), average E/e’ ratio >14, LAVI >34 mL/m^2^, and TR Vmax >2.8 m/s. Definite diastolic dysfunction requires more than half of available parameters exceeding these cutoffs, while indeterminate diastolic function occurs when fewer than half meet the criteria [19]. A previous study found that among patients with functionally insignificant coronary stenosis, those with indeterminate diastolic function exhibited a significantly higher risk of cardiovascular death relative to their counterparts without indeterminate diastolic function [19]. Additionally, among patients with impaired LVEF, indeterminate diastolic function was associated with increased cardiovascular-related mortality [20]. Oh *et al*. [21] introduced a comprehensive algorithm that incorporated four key variables: septal and lateral e’, the mean E/e’ ratio, LAVI, and TR Vmax. A notable feature of this algorithm is its exclusive use of septal TDI measurements, along with specific threshold values: septal e’ <7 cm/s and septal E/e’ >15. This approach employed stringent criteria, requiring the presence of at least 3 out of 4 variables as a preliminary filter. Subsequently, patients were categorized based on the E/A ratio: those with fewer than 3 initial variables were classified as having normal diastolic function if the E/A ratio was >0.8, or as having grade I diastolic dysfunction if the E/A ratio was ≤0.8. For patients with 3 or more initial variables, grade II diastolic dysfunction was assigned if the E/A ratio was <2, and grade III diastolic dysfunction if the E/A ratio was ≥2. Patients with only 2 initial variables were labeled as having undetermined diastolic function [21]. There is a heterogeneity in the diagnostic parameters for LVDD among different studies. This variation is attributed to multiple factors, such as diverse study populations, distinct echocardiographic methodologies, and varying criteria for defining LVDD. For example, while some studies utilize E/e’ ratio as a primary diagnostic parameter, others may employ LAVI or E/A ratio, thereby contributing to inconsistencies in the diagnosis and classification of LVDD [22, 23, 24].

### 2.2 Cardiac Magnetic Resonance

Cardiac magnetic resonance imaging (CMR) is a noninvasive technique that provides precise assessment of cardiac morphology and function. Recently, CMR has emerged as a crucial tool for evaluating LV diastolic function. It provides accurate characterization of LV volume throughout the cardiac cycle, allowing estimation of parameters such as peak filling rate and time to peak filling, both of which are indicative of diastolic function [11, 16]. Additionally, CMR permits the evaluation of LA volume and function, which is crucial for assessing LV diastolic function [25]. It enables precise measurement of LA function, encompassing conduit, pump, and reservoir functions, as well as temporal variations in LA volume [25]. Furthermore, CMR can provide hemodynamic analysis and tissue velocity comparable to via echocardiography. Importantly, mitral valve inflow velocities (E and A waves) and pulmonary vein flow assessed by CMR demonstrate strong correlation with echocardiographic measurements [26, 27]. Strain rate, a pivotal parameter for assessing LV diastole and recovery, is inversely correlated with relaxation time constant, tau [28]. Parameters related to torsion and untwisting are critical for assessing the relaxation and restorative forces of the LV [29]. Strain, strain rate, and torsional and untwisting parameters, can be derived from CMR using three different approaches: tagging, feature tracking, and phase-contrast CMR [29]. In addition, extracellular volume, a marker of fibrosis, has been found to markedly correlate with the load-independent passive LV stiffness constant [30]. T1 mapping imaging combined with feature tracking permits the measurement of extracellular volume, reflecting the stiffness parameter linked to diastolic function, thereby allowing the assessment of diastolic dysfunction [31]. Notably, even though CMR can evaluate diastolic function from multiple perspectives, such as cardiac morphology and function, hemodynamics, and tissue characteristics, which aids in detecting the progression of diastolic dysfunction, its clinical applicability may be limited by lengthy scanning duration and relatively high cost.

## 3. The Differences Between Epicardial and Pericardial Adipose Tissue

**Fig. 1.**
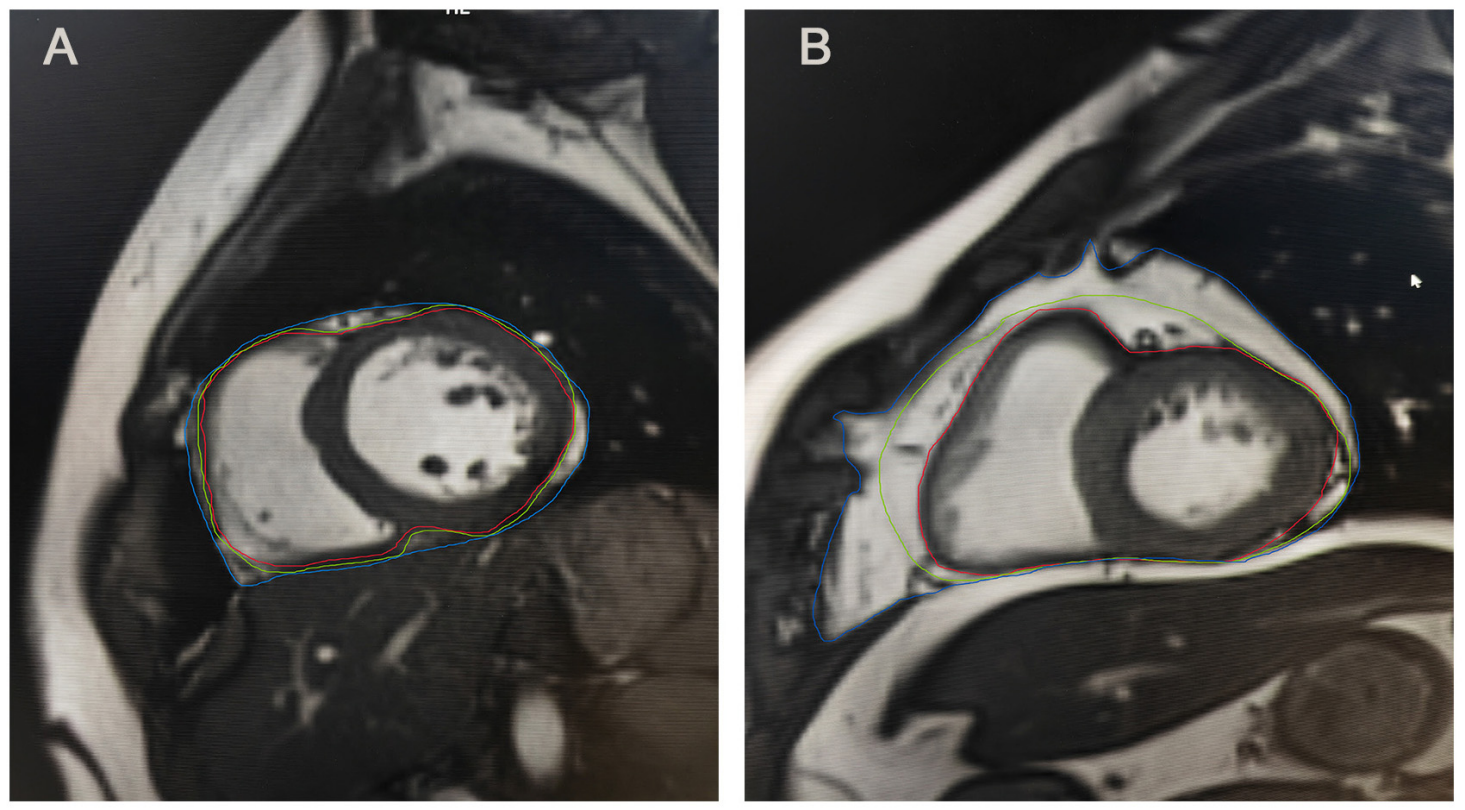
**Epicardial and pericardial adipose tissue on MRI**. (A) Epicardial and pericardial adipose tissue on MRI of individual A. (B) Epicardial and pericardial adipose tissue on MRI of individual B. Individual B exhibited a greater amount of pericardial adipose tissue and epicardial adipose tissue compared to individual A. Epicardial adipose tissue is located between the red and green lines, whereas pericardial adipose tissue is situated between the green and blue lines. MRI, magnetic resonance imaging.

Despite their anatomical proximity, EAT and pericardial adipose tissue (PAT) exhibit fundamental anatomical, embryological, and functional differences. Anatomically, EAT resides between the myocardium and the visceral pericardium, while PAT lies anterior to EAT, situated between the visceral and parietal pericardial layers [32]. Embryologically, EAT adipocytes originate from the splanchnopleuric mesoderm, sharing an origin with mesenteric and omental adipocytes [33]. PAT, in contrast, derives from the primitive thoracic mesenchyme, which differentiates into the parietal pericardium and thoracic wall [33]. Their vascular supply also differs: EAT is supplied by coronary arteries, whereas PAT receives blood from non-coronary sources [33]. Moreover, unlike EAT, PAT lacks a shared microvascular bed with the myocardium [34]. Consequently, EAT and PAT exhibit distinct metabolic and physiological characteristics [35], with EAT demonstrating a stronger association with cardiovascular and metabolic diseases than PAT [36]. In echocardiographic measurements, EAT can be recognized as the echo-free space between the outer wall of the myocardium and the visceral layer of the pericardium [36]. Meanwhile, the PAT thickness can be delineated as the hypoechoic space anterior to the EAT and parietal pericardium, which remains relatively stable and does not deform substantially during the cardiac cycle [36]. On CMR (Fig. 1), EAT is characterized as the adipose tissue between the myocardium and the visceral pericardium, and PAT as adipose tissue external to the parietal pericardium [37].

## 4. Physiology of EAT

EAT is a fat depot situated between the myocardium and the epicardium, sharing a similar origin with visceral fat, both derived from the splanchnopleuric mesoderm [38]. In healthy state, EAT covers approximately 80% of heart surface and accounts for nearly 15% to 20% of the whole cardiac volume [39]. It is distributed along the main branches of the coronary arteries, with the majority found in the atrioventricular and interventricular grooves [7]. Due to the absence of muscle fascia, EAT is in direct contact with the myocardium and coronary arteries, sharing a common microcirculation with the myocardium, which facilitates crosstalk between EAT and the myocardium [7]. Microscopically, EAT predominantly comprises adipocytes but also includes inflammatory, nerve, vascular, and stromal cells [7].

**Fig. 2.**
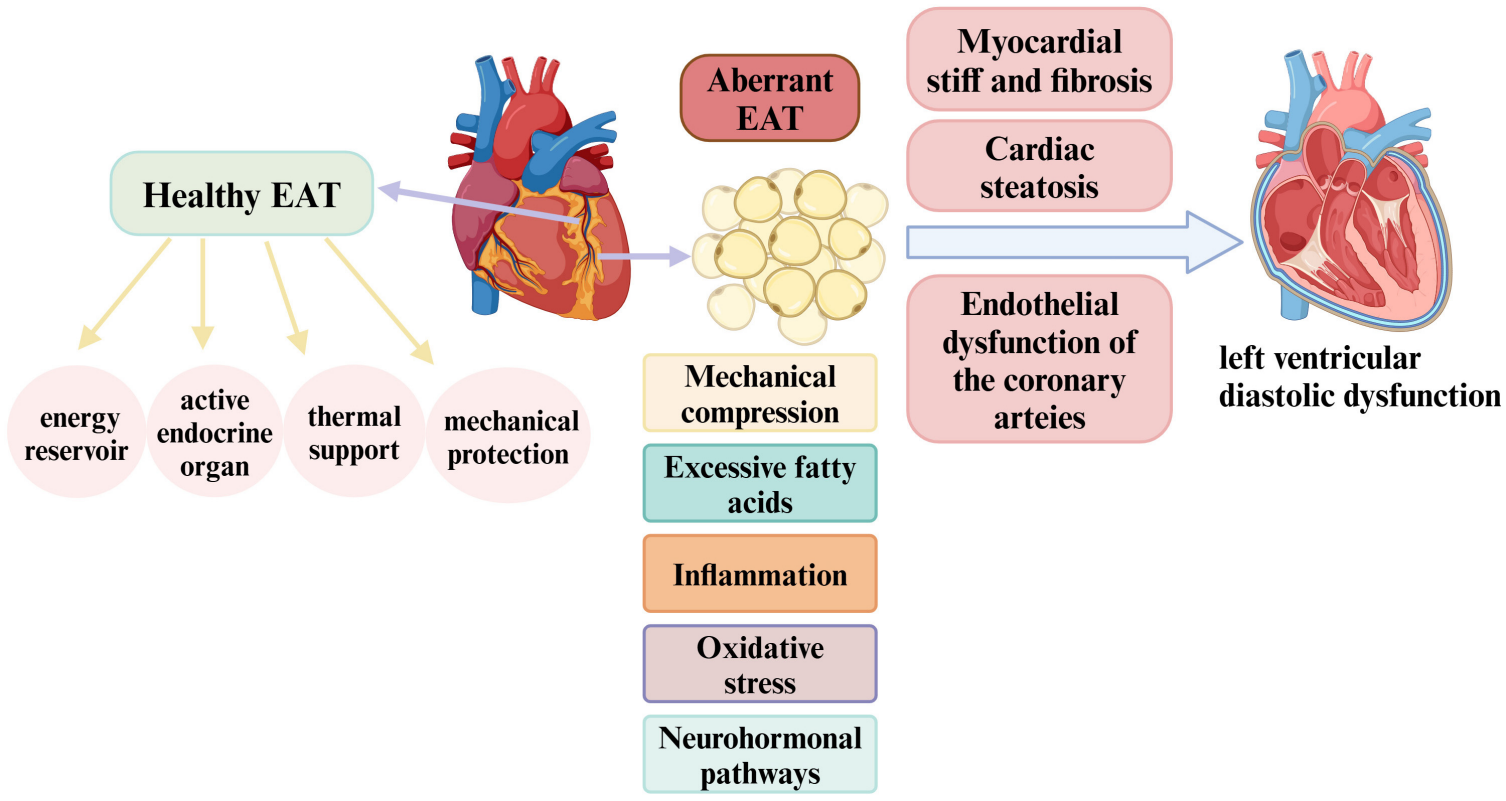
**Physiology of EAT and its role in the pathophysiology of left ventricular diastolic dysfunction**. In a healthy state, EAT serves as an energy reservoir that supports cardiac energy metabolism and functions as an active endocrine organ, modulating the balance between anti-inflammatory and pro-inflammatory responses. Additionally, EAT provides thermal support and mechanical protection to the heart. However, EAT enlargement may contribute to left ventricular diastolic dysfunction development through several mechanisms, including mechanical compression, inflammation, excessive free fatty acid production, oxidative stress, and neurohormonal pathways. These processes can induce myocardial cell stiffness, endothelial dysfunction in the coronary arteries, myocardial fibrosis, and cardiac steatosis, ultimately leading to left ventricular diastolic dysfunction. EAT, epicardial adipose tissue. Created with https://www.biorender.com.

Under physiological conditions, EAT plays protective roles in cardiac metabolism (Fig. 2). EAT maintains the homeostasis of free fatty acids, which are responsible for 60%–70% of energy production in cardiac energy metabolism [40, 41]. *In vitro* studies have demonstrated that EAT takes up and releases free fatty acids more rapidly compared with other adipose tissues [40, 42]. This characteristic enables EAT to serve as an energy reservoir, supplying free fatty acids to myocardial cells through direct diffusion, coronary circulation, and the expression of fatty acid transporters [7, 43]. Moreover, EAT functions as a buffer to protect the myocardium and coronary arteries from lipotoxicity by absorbing and dissolving excess fatty acids from the coronary circulation [42]. As an active endocrine organ, EAT regulates the balance between anti-inflammatory and pro-inflammatory responses through the secretion of various bioactive molecules, including adiponectin, leptin, interleukin (IL)-6, IL-8 and tumor necrosis factor-α (TNF-α) [43, 44]. In addition, EAT can provide direct thermal support to the myocardium during ischemia or hypoxia, possibly due to the high expression of uncoupling protein-1, an indicator of brown adipose tissue [45]. However, the mechanisms underlying the regulation of thermogenesis in EAT are not yet fully understood. Furthermore, owing to its elasticity and compressibility, EAT provides mechanical support, shielding the coronary arteries from excessive distortion and compression resulting from arterial pulsation and myocardial contraction [40].

## 5. Possible Mechanisms of EAT Implicated in the Development of LVDD

**Fig. 3.**
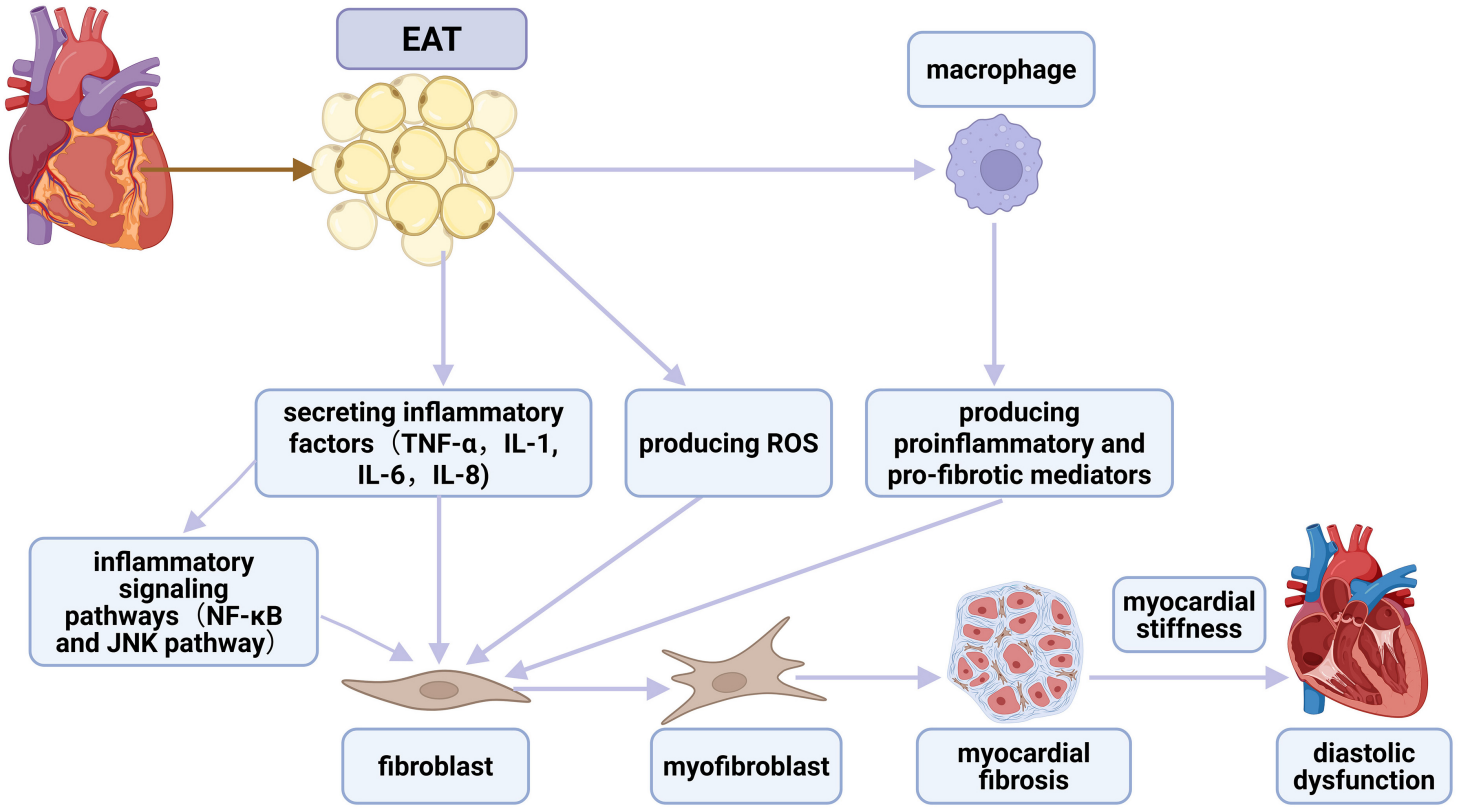
**The association between EAT, inflammation, fibrosis, and diastolic dysfunction**. EAT secretes inflammatory cytokines that directly activate cardiac fibroblasts, promoting their differentiation into myofibroblasts. This process is further facilitated by inflammatory signaling pathways, notably NF-κB and JNK. Additionally, EAT produces increased levels of ROS, promoting myocardial fibrosis. Moreover, pro-inflammatory macrophages infiltrating EAT also contribute by secreting pro-inflammatory and pro-fibrotic mediators. These mediators drive further fibroblast-to-myofibroblast differentiation. Collectively, these processes induce myocardial stiffness, ultimately contributing to the development and progression of diastolic dysfunction. EAT, epicardial adipose tissue; NF-κB, nuclear factor kappa-B; JNK, c-Jun N-terminal kinase; ROS, reactive oxygen species; IL, interleukin; TNF-α, tumor necrosis factor-α. Created with https://www.biorender.com.

EAT may be involved in the pathological processes leading to LVDD through multiple mechanisms (Fig. 2). Firstly, the enlargement of EAT could directly compress the myocardium, which may restrict cardiac expansion, resulting in increased ventricular filling pressures and impaired diastolic function [41]. This phenomenon was illustrated in the Framingham Heart Study, which found that obese individuals with elevated pericardial and/or thoracic fat exhibited diastolic impairment, even in the absence of LV hypertrophy [46]. Secondly, dysfunctional EAT fosters the proinflammatory milieu of the heart by releasing more pro-inflammatory cytokines, attributable to macrophage activation and adipocyte enlargement. Classical pro-inflammatory adipokines, such as IL-6, TNF-α, and IL-1β are prominently expressed in enlarged EAT and adjacent myocardial tissues [47]. These inflammatory mediators contribute to myocardial cell stiffness (Fig. 3), endothelial dysfunction of the coronary arteries, and myocardial fibrosis, subsequently facilitating diastolic dysfunction development [48, 49]. Thirdly, excessive fatty acids overflowing from overnourished EAT directly infiltrate myocardium owing to the absence of a barrier between EAT and myocardium. This process can be detrimental to the heart, as triglycerides accumulation in cardiomyocytes could cause impaired contractility and cardiac dysfunction, evidenced in animal models of lipotoxicity [50]. Clinical studies have demonstrated a positive correlation between EAT and myocardial fat accumulation [51], with increased intramyocardial fat content correlating with parameters of LVDD [52]. In addition, EAT can produce elevated levels of reactive oxygen species, possibly associated with increased expression of hypoxia-inducible factor-1, which damages the myocardium and coronary vessels [53]. Neurohormonal pathways may also play a role in the development of impaired diastolic function. Evidence suggests that EAT is involved in the synthesis of catecholamines, which is correlated with neurohormonal activation and myocardial damage [54]. Notably, in patients with heart failure, it has been observed that following isoproterenol treatment, molecules released from EAT were implicated in inflammatory responses and extracellular matrix [55].

## 6. Measurement of EAT

EAT quantification employs several imaging modalities, with echocardiography, computed tomography (CT), and magnetic resonance imaging (MRI) being the predominant techniques. Initial echocardiographic visualization of EAT thickness was achieved in 2003 using standard two-dimensional imaging at the right ventricular free wall, establishing it as a novel cardiovascular risk indicator [56, 57]. Echocardiography is characterized by several key advantages for the measurement of EAT, such as low cost, accessibility, and non-invasiveness. However, echocardiography is limited to assessing the thickness of EAT at particular locations and cannot measure the EAT volume. Considering the heterogeneous distribution of EAT in patients, EAT thickness serves as a less dependable metric relative to EAT volume. Additionally, echocardiography is an operator-dependent modality, exhibiting inferior reproducibility and accuracy compared with CT-based methods. CT enables assessment of both EAT thickness and volume. The CT attenuation value of EAT typically ranges from –190 to –30 Hounsfield units (HU), allowing clear differentiation from myocardium and pericardium [58]. Although three-dimensional reconstruction software can be used to measure EAT volume, CT applications for long-term follow-up are constrained by some limitations, including the potential impact from arrhythmias, tachycardia, and calcifications, as well as inherent radiation exposure. Moreover, the semi-automatic quantification of EAT volume using three-dimensional reconstruction software may introduce measurement variability. MRI is currently considered the gold standard for measuring EAT, overcoming key limitations of other modalities: its superior spatial resolution addresses echocardiography’s resolution constraints, while radiation-free acquisition avoids CT-related risks [59]. However, clinical MRI implementation faces challenges including prolonged scan times, high costs, and patient-specific limitations (obesity, claustrophobia).

## 7. The Relationship Between EAT and LVDD in Clinical Conditions

### 7.1 Association Between EAT and LVDD in Patients With Cardiovascular Diseases

#### 7.1.1 Coronary Artery Diseases

In populations with coronary artery diseases, increased EAT has been proposed to be related to LVDD (Table 1, Ref. [9, 10, 22, 60, 61, 62, 63, 64, 65, 66, 67, 68, 69, 70, 71, 72, 73, 74, 75]). In this regard, Fontes-Carvalho *et al*. [60] have demonstrated that higher EAT volume independently correlated with worse LV diastolic function in patients after myocardial infarction. Similarly, Hachiya *et al*. [61] have revealed a significant association of EAT and LVDD in 134 patients with known or suspected coronary artery diseases. Additionally, a correlation between EAT and diastolic function has been observed even in patients with ischemia or typical chest pain but without obstructive coronary artery diseases [62]. EAT volume has emerged as a robust predictor of LVDD in patients with chronic coronary syndrome and preserved LVEF, whereas the burden of coronary atherosclerosis itself showed no independent association with LVDD [10]. However, further research is required to determine whether the role of EAT persists independent of myocardial ischemia and coronary microvascular obstruction.

**Table 1.** **Overview of recent clinical studies exploring the role of epicardial adipose tissue in left ventricular diastolic function**.

Study	Participants	Imaging method	Major findings
Hsu *et al*. [9], 2025	Prediabetes (n = 82)	CMR for EAT volume	EAT volume was significantly elevated in prediabetes and diabetes
Observational, cross-sectional	Diabetes (n = 79)	CMR and echocardiography for diastolic function	EAT was associated with cardiac structure and diastolic function in prediabetes
	Normal glucose tolerance (n = 209)		
Song *et al*. [22], 2022	T2DM (n = 116)	Echocardiography for EAT thickness and diastolic function	EAT thickness was independently associated with E/e’
Observational, cross-sectional			
Christensen *et al*. [65], 2019	T2DM (n = 770)	Echocardiography for EAT thickness, systolic function and diastolic function	EAT thickness was higher in T2DM patients than controls
Observational, cross-sectional	Controls (n = 252)		EAT was correlated with reduced diastolic function
Zhu *et al*. [66], 2023	T2DM (263 male and 279 female)	Cardiac computed tomographic for EAT volume	EAT volume was higher in men than in women
Observational, cross-sectional		Echocardiography for diastolic function	EAT volume was considerably related to diastolic function in both sexes
Ahmad *et al*. [67], 2022	T1DM (n = 50)	Echocardiography for EAT thickness and diastolic function	EAT thickness was significantly increased in T1DM children compared with controls
Observational, cross-sectional	Controls (n = 50)		T1DM children have subclinical LVDD associated with increased EAT thickness
Iacobellis *et al*. [68], 2007	Morbidly obese subjects (n = 30)	Echocardiography for EAT thickness and diastolic function	Morbidly obese subjects had significantly higher EAT thickness and lower diastolic filling parameters than controls
Observational, cross-sectional	Controls (n = 20)		EAT thickness was significantly correlated with impairment in diastolic filling in morbidly obese subjects
Alp *et al*. [69], 2014	Obese children (n = 500)	Echocardiography for EAT thickness and cardiac function	Obese children exhibit early subclinical systolic and diastolic dysfunctions correlated with the increase of EAT thickness
Observational, cross-sectional	Controls (n = 150)		
Chin *et al*. [70], 2023	Severe obesity subjects (n = 186)	Echocardiography for EAT thickness and cardiac function	Increased EAT thickness in obese individuals without known cardiac disease was independently related to subclinical cardiac dysfunction
Observational, cross-sectional			
Iacobellis *et al*. [71], 2008	Severe obese subjects (n = 20)	Echocardiography for EAT thickness and diastolic function	EAT thickness decreased after weight loss
Interventional, longitudinal			Changes in EAT are significantly associated with obesity-related alterations in cardiac morphological and functional during weight loss
Ran *et al*. [72], 2024	Cushing’s syndrome (n = 86)	Non-contrast chest CT for EAT volume	Cushing’s syndrome correlated with marked accumulation EAT and prevalence of LVDD
Observational, cross-sectional	Controls (n = 86)	Echocardiography for diastolic function	EAT volume was an independent risk factor for LVDD in Cushing’s syndrome patients
Fontes-Carvalho *et al*. [60], 2014	Patients after myocardial infarction (n = 225)	CT for EAT volume	EAT volume was higher in patients with LVDD
Observational, cross-sectional		Echocardiography for diastolic function	Increasing EAT volume was independently associated with worse LV diastolic function
Hachiya *et al*. [61], 2014	Patients with known or suspected coronary artery disease (n = 134)	CT for EAT volume	EAT was linked to LVDD in patients with known or suspected coronary artery disease
Observational, cross-sectional		Echocardiography for diastolic function	
Topuz and Dogan [62], 2017	Coronary artery disease (n = 85)	Echocardiography for EAT thickness and diastolic function	EAT thickness was markedly associated with LVDD in subjects with normal coronary artery
Observational, cross-sectional	Non-significant coronary artery disease (n = 82)		
	Normal coronary artery (n = 83)		
Ishikawa *et al*. [10], 2024	Chronic coronary syndrome and preserved left ventricular ejection fraction (n = 314)	Coronary computed tomographic angiography for EAT volume	EAT volume index was a robust predictor of LVDD
Observational, cross-sectional		Echocardiography for diastolic function	No independent association found between coronary atherosclerotic disease burden and LVDD
Çetin *et al*. [63], 2013	Hypertensive patients with normal systolic function (n = 127)	Echocardiography for EAT thickness and diastolic function	EAT was significantly increased in patients with high grades of diastolic dysfunction
Observational, cross-sectional			EAT served as an independent predictor of all diastolic dysfunction parameters
Turak *et al*. [64], 2013	Newly diagnosed essential hypertension (60 patients with normal diastolic function and 75 patients with LVDD)	Echocardiography for EAT thickness and diastolic function	EAT thickness was markedly elevated in patients with LVDD
Observational, cross-sectional			Increased EAT thickness was significantly linked to impaired LV diastolic function in patients with newly diagnosed essential hypertension
Yang *et al*. [73], 2022	Obese adolescents (n = 276)	Echocardiography for EAT thickness and diastolic function	EAT highly correlated with reduction in LV diastolic function in adolescents
Observational, cross-sectional			
Cho *et al*. [74], 2024	Subjects with preserved LV systolic function (n = 749)	Echocardiography for EAT thickness, LV structural parameters, and LV functional variables	Compared with visceral adipose tissue or subcutaneous adipose tissue, EAT is the most critical adipose tissue influencing LV geometric and functional changes
Observational, cross-sectional		CT for visceral adipose tissue and subcutaneous adipose tissue	Thicker EAT was related to concentric remodeling and abnormalities in relaxation
Lin *et al*. [75], 2013	Patients undergoing peritoneal dialysis (65 participants with LVDD, 84 subjects without LVDD)	Echocardiography for EAT thickness and diastolic function	EAT rather than visceral fat was an independent risk factor for LVDD in patients undergoing peritoneal dialysis
Observational, cross-sectional		CT for visceral adipose tissue and subcutaneous adipose tissue	

CMR, cardiovascular magnetic resonance; CT, computed tomography; EAT, epicardial adipose tissue; LV, left ventricular; LVDD, left ventricular diastolic dysfunction; T1DM, type 1 diabetes mellitus; T2DM, type 2 diabetes mellitus.

#### 7.1.2 Hypertension

Several studies have explored the relationship between EAT amount and LV diastolic function in hypertensive patients. Çetin *et al*. [63] have demonstrated that increased EAT is significantly associated with diastolic dysfunction parameters, independent of abdominal obesity, in untreated hypertensive patients. Similarly, another study has identified EAT thickness as an independent predictor of LVDD in patients with newly diagnosed essential hypertension [64]. It is important to note, however, that hypertension itself remains a major, well-established risk factor for LVDD, primarily driven by pressure overload, inflammation, myocardial ischemia, and other mechanisms.

### 7.2 Association Between EAT and LVDD in Patients With Metabolic Disorders

#### 7.2.1 Diabetes

EAT expansion is closely associated with LVDD in diabetic populations. Song *et al*. [22] have reported that thickened EAT was linked to impaired LV diastolic function in patients with type 2 diabetes mellitus (T2DM). A cross-sectional study involving 770 T2DM patients found a correlation between EAT and reduced diastolic function, as evidenced by E/A ratio, myocardial velocity (lateral e’), and lateral E/e’ ratio [65]. EAT volume was higher in male T2DM patients compared to female T2DM patients and showed a significant association with diastolic function in both sexes after adjusting for risk factors [66]. EAT also adversely impacts diastolic function in prediabetes. Hsu *et al*. [9] have observed that elevated EAT in patients with prediabetes could be implicated in adverse changes in cardiac structure and diastolic function, potentially responsible for the early onset of diabetic cardiomyopathy. Likewise, children with type 1 diabetes mellitus (T1DM) exhibit significantly greater EAT thickness than healthy controls, which is associated with subclinical LVDD and vascular endothelial dysfunction [67].

#### 7.2.2 Obesity

The excessive accumulation of EAT may be involved in early stages of cardiac dysfunction in individuals with obesity. Iacobellis *et al*. [68] first established the relationship between increased EAT thickness and impaired diastolic filling in morbidly obese patients. Furthermore, another study has indicated that obese children exhibit early subclinical diastolic dysfunction associated with greater EAT thickness [69]. A similar association exists in obese individuals without established heart disease; Chin *et al*. [70] have found that increased EAT thickness in obese subjects without known cardiac issues independently correlated with subclinical cardiac dysfunction, suggesting EAT may serve as an early marker for cardiac impairment. In severe obesity, alterations in diastolic function closely parallel variations in EAT. Specifically, changes in the E/A ratio following weight loss were considerably related to modifications in EAT [71]. Additionally, centripetal obesity induced by excess cortisol also presented a notable accumulation of EAT, demonstrating a strong correlation with LVDD [72].

Overall, increasing evidence has indicated that EAT was elevated in diabetic population and obese subjects. In these individuals, higher EAT volume adversely impacts cardiac diastolic function. However, further investigations are required to determine whether T2DM and obesity exacerbate the pathogenic potential of EAT and to elucidate the role of EAT in early diastolic dysfunction in these patients.

## 8. Targeting EAT as a Potential Strategy for Improving Diastolic Function

**Fig. 4.**
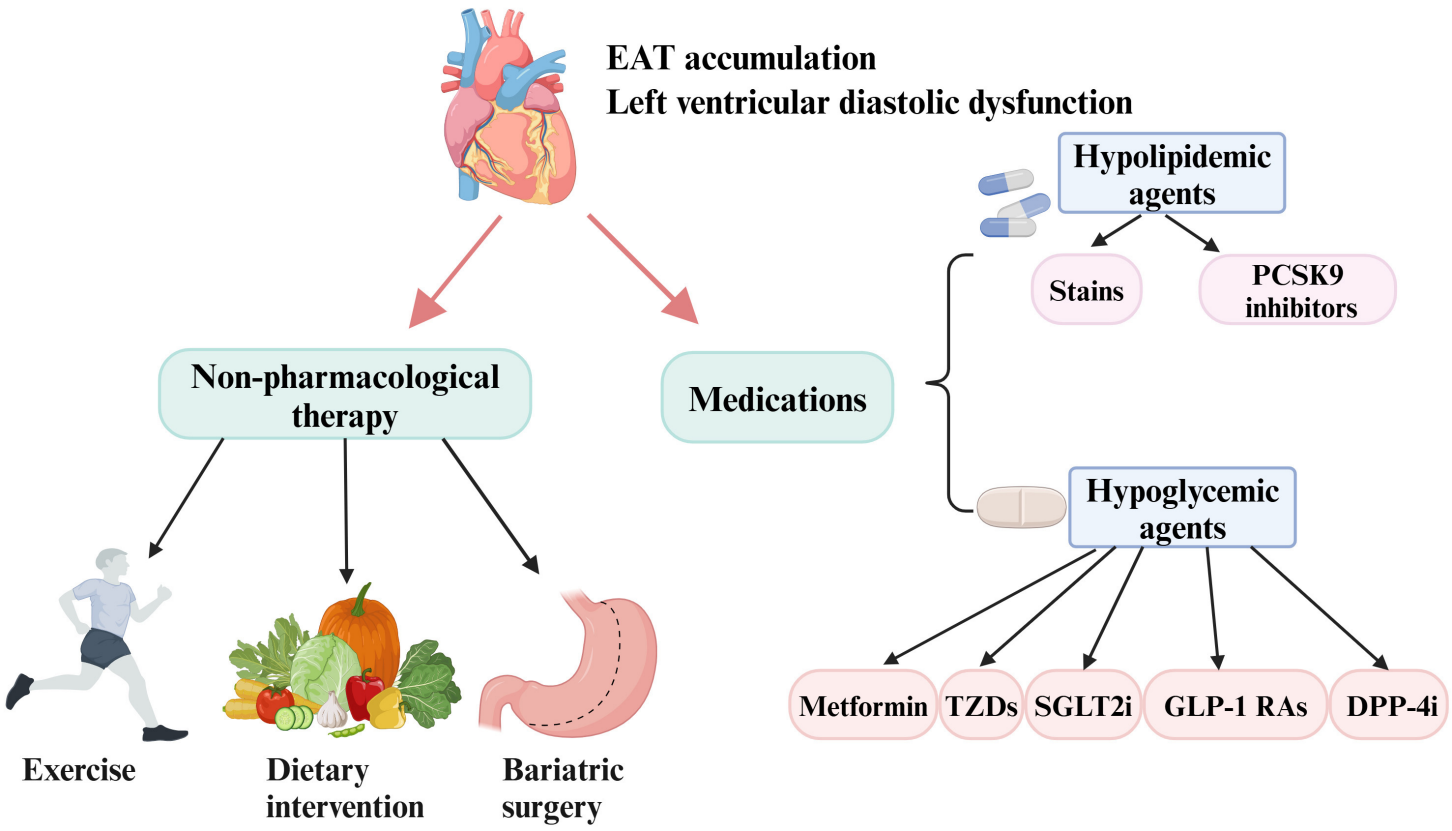
**Current interventions aimed at reducing EAT and improving diastolic function include pharmacological and non-pharmacological therapies**. Some agents such as metformin, TZDs, SGLT2i, GLP-1 RAs, DPP-4i, and hypolipidemic agents have shown promise in decreasing EAT deposition and ameliorating diastolic function. In addition, lifestyle modifications (exercise and diet) and bariatric surgery have demonstrated beneficial effects in inhibiting EAT expansion and improving diastolic function. EAT, epicardial adipose tissue; PCSK9, proprotein convertase subtilisin/kexin type 9; DPP-4i, dipeptidyl peptidase-4 inhibitors; GLP-1 RAs, glucagon-like peptide-1 receptor agonists; SGLT2i, sodium-glucose cotransporter-2 inhibitors; TZDs, thiazolidinediones. Created with https://www.biorender.com.

Given rapid advancements in research, there is an increasing acknowledgement that EAT may contribute to the pathophysiology of LVDD. Consequently, reducing EAT deposition could be a promising strategy for improving LVDD to prevent heart failure. Although no specific treatments currently target EAT directly, we discuss four potential interventions (Fig. 4) proposed to mitigate EAT expansion and ameliorate diastolic function: medications (Table 2, Ref. [76, 77, 78, 79, 80, 81, 82, 83, 84, 85, 86, 87, 88, 89, 90, 91, 92]), diet, exercise, and bariatric surgery, with a particular focus on pharmaceutical interventions.

**Table 2.** **Medications targeting epicardial adipose tissue**.

Study	Participants	Imaging method	Intervention strategy	Change of EAT
Iacobellis and Gra-Menendez [76], 2020	Patients with T2DM and obesity (n = 100)	Echocardiography for EAT thickness	Dapagliflozin (n = 50): 5 mg/d, 4 weeks; 10 mg/d, 20 weeks	Dapagliflozin treatment resulted in a 20% reduction in EAT. Metformin treatment caused a 7% reduction in EAT.
			Metformin (n = 50): 500 mg to 1000 mg twice daily, 24 weeks	
Ziyrek *et al*. [77], 2019	Patients with newly diagnosed T2DM (n = 40)	Echocardiography for EAT thickness	Metformin (n = 40): 1000 mg twice daily, 3 months	EAT thickness decreased from 5.07 ± 1.33 mm to 4.76 ± 1.32 mm (*p * < 0.001)
Iacobellis *et al*. [78], 2017	Patients with T2DM and obesity (n = 95)	Echocardiography for EAT thickness	Combination of liraglutide (n = 54, 0.6 mg to 1.8 mg once daily) and metformin (500 mg to 1000 mg twice daily), 6 months	EAT decreased from 9.6 ± 2 to 6.2 ± 1.5 mm (*p * < 0.001)
Moody *et al*. [79], 2023	Patients with T2DM (n = 12)	CMR for EAT area	Pioglitazone (n = 12) from 15 mg/day to 45 mg/day, 24 weeks	EAT area decreased from 15.3 ± 3.9 to 14.0 ± 3.9 cm^2^ (*p* = 0.03)
Brandt-Jacobsen *et al*. [80], 2023	Patients with T2DM (n = 90)	PET/CT for cardiac adipose tissue volume	Empagliflozin (n = 38) 25 mg/day, 13 weeks	Cardiac adipose tissue volume decreased 4.8% (*p* = 0.034)
Yagi *et al*. [81], 2017	Patients with T2DM (n = 13)	Echocardiography for EAT thickness	Canagliflozin (n = 13) 100 mg/day, 6 months	EAT thickness decreased from 9.3 ± 2.5 to 7.3 ± 2.0 mm (*p * < 0.001)
Gaborit *et al*. [82], 2021	Patients with T2DM (n = 56)	CMR for EAT volume	Empagliflozin (n = 26) 10 mg/day, 12 weeks	There were no significant changes in EAT volume after 12 weeks of empagliflozin treatment
Macías-Cervantes *et al*. [83], 2024	Patients with acute coronary syndrome (n = 52)	Non-contrast cardiac CT for EAT volume	Dapagliflozin (n = 28) 10 mg/day, 12 months	There was no effect on EAT volume change with the use of dapagliflozin
Bizino *et al*. [84], 2020	Patients with T2DM (n = 50)	MRI for EAT, visceral fat, and subcutaneous fat	Liraglutide (n = 23) 1.8 mg/day, 26 weeks	Liraglutide primarily reduced subcutaneous fat but not visceral fat, or EAT
Zhao *et al*. [85], 2021	Patients with abdominal and obesity T2DM (n = 50)	CMR for EAT thickness	Liraglutide (n = 21), 0.6 mg/day to 1.8 mg/day, 3 months	EAT thickness decreased from 5.0 (5.0–7.0) mm to 3.95 ± 1.43 mm (*p * < 0.001)
Iacobellis *et al*. [86], 2020	Patients with T2DM and obesity (n = 80)	Echocardiography for EAT thickness	Semaglutide (n = 30), up to 1 mg weekly, 12 weeks	EAT thickness decreased in both semaglutide and dulaglutide groups (*p * < 0.001), accounting for a 20% reduction
			Dulaglutide (n = 30), up to 1.5 mg weekly, 12 weeks	There was no EAT reduction in the metformin group
			Metformin (n = 20), 12 weeks	
Dutour *et al*. [87], 2016	Patients with obesity and T2DM (n = 44)	MRI for EAT volume	Exenatide (n = 22), 5 mg twice daily for 4 weeks; then 10 mg twice daily for 22 weeks	The EAT volume decreased by 8.8 ± 2.1% (*p* = 0.003), and this reduction was significantly correlated with weight loss
Lima-Martínez *et al*. [88], 2016	Subjects with T2DM and obesity (n = 26)	Echocardiography for EAT thickness	Sitagliptin/metformin (n = 26) 50 mg/1000 mg twice daily, 24 weeks	EAT decreased significantly from 9.98 ± 2.63 to 8.10 ± 2.11 mm, (*p* = 0.001)
Raggi *et al*. [89], 2019	Postmenopausal women (n = 420)	CT for EAT attenuation	Atorvastatin (n = 194), 80 mg/day, 1 year	Both atorvastatin and pravastatin can considerably reduce EAT attenuation (*p * <0.001)
			Pravastatin (n = 226), 40 mg/day, 1 year	
Park *et al*. [92], 2010	Patients with coronary artery stenosis underwent percutaneous coronary intervention (n = 145)	Echocardiography for EAT thickness	Atorvastatin (n = 82), 20 mg/day for 6–8 months	EAT thickness decreased from 4.08 ± 1.37 to 3.76 ± 1.29 mm with statin treatments (*p * < 0.001)
			Simvastatin/ezetimibe (n = 63), 10/10 mg daily for 6–8 months	
Alexopoulos *et al*. [90], 2013	Hyperlipidemic post-menopausal women (n = 420)	CT for EAT volume	Atorvastatin (n = 194), 80 mg/day, 12 months	The percentage reduction in EAT was significantly greater in atorvastatin group compared to those treated with pravastatin (median reduction: 3.38% vs. 0.83%, *p* = 0.025)
			Pravastatin (n = 226), 40 mg/day, 12 months	
Parisi *et al*. [91], 2019	Aortic stenosis patients (n = 193)	Echocardiography for EAT thickness	Statin therapy (n = 87) for a duration ranged from 3 to 72 months	Statin therapy was related to lower EAT thickness (*p * < 0.0001)

CMR, cardiovascular magnetic resonance; CT, computed tomography; EAT, epicardial adipose tissue; T2DM, type 2 diabetes mellitus; PET/CT, positron emission tomography/computed tomography; MRI, magnetic resonance imaging.

### 8.1 Medications

#### 8.1.1 Hypoglycemic Agents

8.1.1.1 Metformin

The effects of metformin on cardiac function have been extensively investigated, with recent studies also examining its influence on EAT. Available evidence indicates that metformin positively modifies body composition and reduces visceral adiposity [93]. In particular, metformin treatment can decrease EAT thickness in patients with T2DM [76, 77]. Sardu *et al*. [94] have reported that metformin could mitigate pericoronary fat inflammation, thereby improving prognosis in prediabetic patients with acute myocardial infarction. In terms of diastolic dysfunction, metformin treatment has been linked to improved diastolic function in individuals with T2DM and metabolic syndrome [95, 96]. In a mouse model, metformin has been proposed to enhance diastolic function by increasing titin compliance [97]. Nevertheless, Iacobellis *et al*. [78] have shown that metformin failed to reduce EAT thickness after 3–6 months of treatment in T2DM patients. In addition, metformin was not effective in ameliorating diastolic function in patients presenting with ST-elevation myocardial infarction and hypertensive patients with T2DM [98, 99]. Overall, the effects of metformin on EAT and diastolic function remain controversial, highlighting the need for further research to provide more robust evidence.

8.1.1.2 Thiazolidinediones

Thiazolidinediones (TZDs), such as pioglitazone and rosiglitazone, are commonly employed in T2DM management. Accumulating evidence indicates that pioglitazone can improve diastolic function in individuals with T2DM. For instance, a study has demonstrated a 24-week treatment with pioglitazone significantly improved diastolic function in patients with well-controlled T2DM [100]. Consistent with these findings, Tsuji *et al*. [101] observed that pioglitazone therapy positively affected diastolic function in prediabetic stage of type II diabetic rats. Furthermore, in hypertensive patients, pioglitazone has been shown to ameliorate diastolic function [102], a finding that was corroborated in a rat model of hypertension induced by angiotensin II infusion [103]. Importantly, the E/A ratio in obese subjects with metabolic syndrome increased following pioglitazone treatment [104]. This cardioprotective effect may be mediated through reductions in EAT, as evidenced by observations in a cohort of 12 T2DM patients without cardiovascular diseases [79]. Notwithstanding, it is important to note that the use of TZDs increases the risk of developing congestive heart failure [105, 106].

8.1.1.3 Sodium-Glucose Cotransporter-2 Inhibitors

Sodium-glucose cotransporter-2 inhibitors (SGLT2i) have shown cardiovascular benefits, including reductions in heart failure and cardiovascular mortality [107, 108]. These effects may be attributed to both their hypoglycemic and non-hypoglycemic mechanisms, particularly through the modulation of EAT. Multiple studies have reported that SGLT2i, such as empagliflozin [80], dapagliflozin [109], and canagliflozin [81], can effectively decrease EAT accumulation in T2DM patients. Canagliflozin can reduce EAT thickness independent of its glucose-lowering effects [81]. Dapagliflozin has been found to enhance glucose uptake in EAT, decrease the secretion of pro-inflammatory chemokines, and promote EAT differentiation [110]. Additionally, empagliflozin has been described to suppress the differentiation and maturation of human epicardial preadipocytes and modulate the secretion of inflammatory factors from EAT [111]. However, there are also studies with contrary results, for example, clinical studies have indicated that empagliflozin failed to significantly alter EAT in patients with T2DM [82], and dapagliflozin exhibited no effect on EAT volume in patients with acute coronary syndrome [83]. Concerning LV diastolic function, Shim *et al*. [112] observed that remarkable improvement in diastolic function in T2DM patients following treatment with dapagliflozin. The proposed mechanism involves targeting coronary endothelium, reducing inflammation, and attenuating cardiac fibrosis through the regulation of serum and glucocorticoid-regulated kinase 1 signaling, supported by findings from animal model studies [113, 114]. Although empagliflozin has been demonstrated to ameliorate diastolic function in rat models [115, 116], Rai *et al*. [117] indicated a 6-month treatment with empagliflozin had no significant impact on LV diastolic function in patients with T2DM and coronary artery disease. Furthermore, it is plausible that additional mechanisms contribute to the improvement of diastolic function mediated by SGLT2i. SGLT2i exert therapeutic effects by targeting renal proximal tubule sodium-glucose cotransporter 2 [118], leading to increased natriuresis and osmotic diuresis. This reduces blood pressure and cardiac workload, collectively improving cardiodynamic hemodynamics and diastolic function [118]. Moreover, SGLT2i induce weight loss, which may be associated with improved diastolic function [119]. In conclusion, it is still a matter of debate whether SGLT2i play an essential role in modulating EAT deposition and improving diastolic function, and further investigations are warranted.

8.1.1.4 Glucagon-Like Peptide-1 Receptor Agonists

Glucagon-like peptide-1 receptor agonists (GLP-1 RAs), commonly used in treating T2DM and obesity, offer cardiovascular protection beyond their glucose-regulating effects. While several studies have found that liraglutide failed to reduce EAT accumulation in T2DM patients, primarily affecting visceral or subcutaneous fat [84, 120], others document EAT reductions with GLP-1 RAs, including liraglutide [85], semaglutide [86], dulaglutide [86], and exenatide [87]. A meta-analysis revealed a marked reduction in EAT in T2DM patients treated with GLP-1 RAs [121]. Additionally, GLP-1 RAs appeared to be more effective in EAT reduction than SGLT2 inhibitors and statins [122]. The presence of GLP-1 receptors in EAT further supports the hypothesis that GLP-1 RAs may exert a direct influence on EAT deposition [123, 124]. Targeting the GLP-1 receptor in EAT may diminish local adipogenesis, enhance fat utilization, and promote fat browning [123, 124]. Saponaro *et al*. [125] reported that 6 months of liraglutide treatment considerably improved diastolic function in T2DM patients. Moreover, liraglutide treatment has been demonstrated to substantially improve diastolic function compared to oral antidiabetic medications [126]. A randomized controlled trial demonstrated that exenatide mitigated diastolic dysfunction in individuals with T2DM [127]. Yagi *et al*. [128] showed that liraglutide-induced diastolic improvement was predominantly dependent on body weight reduction. Consequently, GLP-1 RAs may inhibit EAT accumulation and potentially benefit patients with LVDD. Nevertheless, the precise mechanisms underlying the improvement in diastolic function associated with GLP-1 RAs remain to be elucidated, specifically whether it is attributable to the reduction of EAT or systemic effects, such as weight loss.

8.1.1.5 Dipeptidyl Peptidase-4 Inhibitors

Dipeptidyl peptidase-4 inhibitors (DPP-4i) are oral anti-diabetic agents that decrease blood glucose levels by augmenting incretin hormone action. There is limited research on the effects of DPP-4 inhibitors on EAT. An observational study involving 26 patients with T2DM and obesity found a significant and rapid reduction in EAT following 24 weeks of sitagliptin treatment, compared to metformin monotherapy [88]. In addition, the impact of DPP-4 inhibitors on diastolic function remains a topic of debate. Nogueira *et al*. [129] reported an improvement in LVDD in 35 T2DM patients after 24 weeks of sitagliptin therapy, suggesting cardioprotective effects of DPP-4i independent of glucose control. Another study found that sitagliptin improved cardiac diastolic dysfunction in diabetic rats [130]. Conversely, a prospective study indicated that while sitagliptin and linagliptin improved blood glucose levels, blood pressure, and proteinuria, they exerted no a considerable impact on diastolic function in T2DM patients [131].

#### 8.1.2 Hypolipidemic Agents

Statins, extensively utilized in the management of dyslipidemia, have also been shown to successfully reduce EAT amount and ameliorate LVDD. Raggi *et al*. [89] have confirmed that reduction in EAT was correlated with treatment using atorvastatin and pravastatin for 1 year in postmenopausal women, independent of their lipid-lowering effects. Intensive lipid-lowering therapy has been found to be more effective in reducing EAT than moderate-intensity therapy [90]. In addition to these effects, statin treatment has been shown to modulate the metabolic activity of EAT and decrease inflammatory mediators secreted by EAT [91], potentially linked to improvements in diastolic function. Animal studies have further suggested that statins may confer benefits for LVDD; for instance, in a rat model of T2DM, moderate lipid lowering with pravastatin prevented diastolic dysfunction [132]. Similarly, long-term atorvastatin treatment ameliorated LVDD in a murine model of obesity [133]. A clinical trial revealed that both atorvastatin and rosuvastatin effectively improved ventricular function and reduced lipid levels in T2DM patients with dyslipidemia, while also modulating inflammation [134]. Besides, atorvastatin was discovered to enhance regional LV diastolic function in patients with coronary artery disease, irrespective of its lipid-lowering effects [135]. Notwithstanding, the extent to which statin therapy alleviates LVDD through reductions in EAT requires validation in additional randomized controlled trials. Proprotein convertase subtilisin/kexin type 9 (PCSK9) inhibitors, a novel class of hypolipidemic agents, have exhibited potential in preventing the expansion of EAT. According to Rivas Galvez *et al*. [136], both evolocumab and alirocumab were proven to be effective in decreasing EAT following a 6-month treatment period. Nonetheless, there is currently insufficient evidence regarding the effects of PCSK9 inhibitors on modulating diastolic function.

### 8.2 Non-Pharmacological Therapy

Other treatment options including lifestyle modification (diet and exercise) and bariatric surgery demonstrate efficacy in reducing EAT and improving diastolic function. Notably, a low-calorie diet in severely obese individuals has exhibited potential effects in reducing EAT and ameliorating diastolic function, with changes in diastolic function correlating with alterations in EAT [71]. Numerous studies have reached conclusions that exercise training can reduce EAT and improve diastolic function [137, 138, 139]. A randomized clinical trial by Christensen *et al*. [138] indicated that both aerobic and resistance exercise can decrease EAT in individuals with abdominal obesity. Moreover, exercise training has been proven successful in ameliorating diastolic function in working-age adults with T2DM [139] and patients with coronary artery diseases [137]. Multiple studies have investigated the impact of bariatric surgery on the reduction of EAT. According to a meta-analysis, bariatric surgeries (laparoscopic sleeve gastrectomy and Roux-en-Y gastric bypass surgery) are capable of decreasing EAT amount [140]. Kurnicka *et al*. [141] found that 6 months after bariatric surgery, there was a notable improvement in LV diastolic function in morbidly obese women. However, current evidence remains insufficient to directly establish that EAT reduction mediates the improvement in diastolic function resulting from these therapies, as they also exert multiple systemic effects, including weight loss, reduced visceral fat, improved blood pressure and enhanced glycemic control. Therefore, further investigations are required to validate these findings.

## 9. Conclusions and Future Directions

LVDD has been recognized as a fundamental pathophysiological mechanism in heart failure. Thus, it is crucial to timely identify diastolic dysfunction using echocardiography or CMR in high-risk populations, including those with cardiovascular diseases and metabolic disorders. EAT, as a modifiable cardiovascular risk factor, may directly contribute to the development of LVDD through mechanisms involving mechanical compression, inflammation, overproduction of free fatty acids, oxidative stress, and neurohormonal pathways. Consequently, modulating EAT accumulation may be a promising therapeutic approach for improving diastolic function. In addition to lifestyle modification, several agents have been shown to ameliorate EAT deposition, such as metformin, TZDs, SGLT2i, GLP-1 RAs, DPP-4i, and lipid-lowering agents. As an alternative for morbid obesity, bariatric surgery can markedly reduce EAT expansion and improve diastolic function. Nonetheless, research on EAT and diastolic function is still subject to several limitations. Firstly, it remains unclear what specific role EAT plays in the pathogenesis of LVDD in diverse clinical settings. Current studies on the relationship between EAT and LVDD primarily focus on T2DM and obesity, while whether EAT extends its influence on LVDD in other conditions warrants further investigation. Secondly, the underlying mechanisms of therapies targeting EAT in alleviating LVDD have not been fully clarified. Further investigations are needed to determine whether the improvement in diastolic dysfunction is directly attributable to EAT reduction, rather than systemic effects such as weight loss. Currently, most interventions are directed towards obesity and T2DM. The applicability of these approaches to other conditions associated with diastolic dysfunction requires further exploration.
